# Monocyte distribution width as a pragmatic screen for SARS-CoV-2 or influenza infection

**DOI:** 10.1038/s41598-022-24978-w

**Published:** 2022-12-13

**Authors:** Oluwakemi Badaki-Makun, Scott Levin, Arnaud Debraine, Benjamin Hernried, Alexandra Malinovska, Aria Smith, Matthew Toerper, Katherine Z. J. Fenstermacher, Taylor Cottle, Malgorzata Latallo, Richard E. Rothman, Jeremiah S. Hinson

**Affiliations:** 1 Department of Pediatrics, Johns Hopkins University School of Medicine, Baltimore, MD USA; 2 Malone Center for Engineering in Healthcare, Johns Hopkins Whiting School of Engineering, 1800 Orleans Street, Baltimore, MD 21287 USA; 3 Department of Emergency Medicine, Johns Hopkins University School of Medicine, Baltimore, MD USA; 4 StoCastic, Baltimore, MD USA; 5 Beckman Coulter, Brea, CA USA; 6 Department of Epidemiology, Johns Hopkins University Bloomberg School of Public Health, Baltimore, MD USA

**Keywords:** Infectious diseases, Biomarkers, Medical research

## Abstract

Monocyte distribution width (MDW) is a novel marker of monocyte activation, which is known to occur in the immune response to viral pathogens. Our objective was to determine the performance of MDW and other leukocyte parameters as screening tests for SARS-CoV-2 and influenza infection. This was a prospective cohort analysis of adult patients who underwent complete blood count (CBC) and SARS-CoV-2 or influenza testing in an Emergency Department (ED) between January 2020 and July 2021. The primary outcome was SARS-CoV-2 or influenza infection. Secondary outcomes were measures of severity of illness including inpatient hospitalization, critical care admission, hospital lengths of stay and mortality. Descriptive statistics and test performance measures were evaluated for monocyte percentage, MDW, white blood cell (WBC) count, and neutrophil to lymphocyte ratio (NLR). 3,425 ED patient visits were included. SARS-CoV-2 testing was performed during 1,922 visits with a positivity rate of 5.4%; influenza testing was performed during 2,090 with a positivity rate of 2.3%. MDW was elevated in patients with SARS-Cov-2 (median 23.0U; IQR 20.5–25.1) or influenza (median 24.1U; IQR 22.0–26.9) infection, as compared to those without (18.9U; IQR 17.4–20.7 and 19.1U; 17.4–21, respectively, *P* < 0.001). Monocyte percentage, WBC and NLR values were within normal range in patients testing positive for either virus. MDW identified SARS-CoV-2 and influenza positive patients with an area under the curve (AUC) of 0.83 (95% CI 0.79–0.86) and 0.83 (95% CI 0.77–0.88), respectively. At the accepted cut-off value of 20U for MDW, sensitivities were 83.7% (95% CI 76.5–90.8%) for SARS-CoV-2 and 89.6% (95% CI 80.9–98.2%) for influenza, compared to sensitivities below 45% for monocyte percentage, WBC and NLR. MDW negative predictive values were 98.6% (95% CI 98.0–99.3%) and 99.6% (95% CI 99.3–100.0%) respectively for SARS-CoV-2 and influenza. Monocyte Distribution Width (MDW), available as part of a routine complete blood count (CBC) with differential, may be a useful indicator of SARS-CoV-2 or influenza infection.

## Introduction

Monocyte distribution width (MDW), a measure of variation in monocyte cell volume, is emerging as a novel indicator of sepsis^1–3^. Monocytes are a subpopulation of circulating leukocytes whose activation in response to infectious stimuli is a key part of the host immune response^4,5^. These mononuclear phagocytes secrete chemokines and cytokines, can differentiate into macrophages and dendritic cells in the presence of appropriate stimuli, and are instrumental in the production of antigen presenting cells^4,5^. Monocyte recruitment is accompanied by dynamic changes in cell morphology that manifest as increased variability in size across the cell population; these changes make MDW a valuable marker for detection of infection and for estimation of disease severity in sepsis^6–8^. However, monocytes are also known to be differentially activated in the presence of viral pathogens, facilitating inflammation and immune targeting, but also serving as reservoirs and aiding in virus dissemination and propagation^9–12^. Thus, MDW may be elevated in viral infections even in the absence of severe disease and may be useful as a screening tool for such infections in the Emergency Department (ED).

The coronavirus disease (COVID-19) pandemic has rapidly become the greatest modern public health emergency with more than 633 million cases and 6.6 million deaths reported worldwide^13^. By December 2020, COVID-19 had become the third leading cause of death in the United States^14,15^. Asymptomatic and pre-symptomatic cases contribute significantly to ongoing transmission of SARS-CoV-2 (the virus that causes COVID 19) and are key drivers of the pandemic^16,17^. Yet, patient symptoms are often used as a prerequisite for testing even in the ED, particularly when demand for testing outpaces supply^18,19^. The complete blood count (CBC) is a frequently ordered laboratory test in the ED and MDW as a component of this has the potential to be used to screen for patients with SARS-CoV-2 infection.

While the current pandemic is caused by a coronavirus, the influenza virus has been a more prominent etiology of epidemics and pandemics in recent decades, having caused severe global pandemics in 1918, 1957–58, 1968, and 2009^20,21^. Influenza is endemic and regularly triggers seasonal global outbreaks resulting in critical illness in as many as five million infected patients and causing more than 500,000 deaths annually^22^. As with SARS-CoV-2, limiting the spread of influenza is key to decreasing morbidity and mortality from seasonal influenza and influenza pandemics, therefore early identification and isolation of minimally symptomatic or asymptomatic patients infected with influenza viruses is also of major importance.

We hypothesized that MDW would perform well as a screen for SARS-CoV-2 and influenza infection. In this study, we measured the sensitivity and specificity of MDW and other leukocyte parameters for SARS-CoV-2 and influenza infection. In addition, we measured the correlation between MDW levels and illness severity in patients with and without SARS-CoV-2 and influenza infection.

## Methods

### Study design and setting

This prospective study was conducted between January 21st, 2020, and July 14th, 2021, in the ED of a quaternary care hospital in Baltimore, MD. The study was performed under a waiver of informed consent given that samples were analyzed as a part of routine care and was approved by the Johns Hopkins School of Medicine Institutional Review Board (IRB). This manuscript follows Standards for Reporting of Diagnostic Accuracy Studies (STARD) guidelines.

### Patient population

All adult patients (aged 18 and over) who had a CBC collected within 6 h of ED arrival as a part of routine clinical care were eligible for the study. Patients were enrolled consecutively during time periods when study team members were present (8–16 h per day). Patients missing a valid MDW (e.g., low sample volume or poor sample quality), patients with MDW sample analyses performed more than 2 h after blood collection, and patients missing other CBC parameters (monocyte percentage, WBC count, NLR) within 6 h of arrival were excluded. A separate analysis was performed in the subgroup of patients who were immunocompromised; these patients were considered to have the highest risk of complications from either infection. Immunocompromise was defined as neutropenia (an absolute neutrophil count of 1.5 × 10^9^/L or lower) on the CBC collected within 6 h of ED arrival and/or the presence of an active immunocompromised state as defined by the Agency for Healthcare Research and Quality (AHRQ)^23^.

### Measurements

Demographics, clinical data (presenting complaints, co-morbidities, vital signs, laboratory), and hospital utilization data were collected from the electronic health record (EHR) system. Presenting complaints were entered from a picklist at ED triage and co-morbidities were mined by grouping diagnostic codes (ICD-10) for active problems available in the EHR at patient presentation^23–25^. MDW was analyzed on a UniCel DxH900 analyzer (Beckman Coulter, Inc), software version 1.0 in potassium ethylenediaminetetraacetic acid (K_2_ EDTA) tubes. A value of greater than 20 Units was defined as abnormal using previously established cutoff criteria^26^. MDW measurement was performed by a study team member blind to patient clinical information. MDW was not reported in the EHR; clinicians were blinded to MDW values while providing care to patients enrolled. Other CBC parameters (monocyte percentage, WBC count and NLR) were measured on a separate hematology analyzer used for routine clinical practice and were available to treating clinicians. An abnormal monocyte percentage was defined as > 10%^27^, an abnormal WBC count was defined as less than 4 × 10^9^/L or greater than 12 × 10^9^/L^28,29^ and an abnormal NLR was defined as greater than 10^30^.

### Outcomes

The primary outcome was viral infection (SARS-CoV-2 or influenza), detected by reverse transcriptase polymerase chain reaction (RT-PCR) assay. All RT-PCR assays were performed on nasopharyngeal samples obtained within 48 h of MDW sample collection. Influenza infection (influenza A or B) was diagnosed using the Xpert Xpress influenza test (Cepheid, Sunnyvale, CA, USA). For the diagnosis of SARS-CoV-2, a variety of RT-PCR platforms were utilized based on availability including the Xpert Xpress SARS-CoV-2 test (Cepheid, Sunnyvale, CA, USA), the RealStar SARS-CoV-2 RT-PCR Kit 1.0 (Altona Diagnostics, Hamburg, Germany), the NeuMoDx SARS-CoV-2 assay (NeuMoDx, Ann Arbor, MI, USA), SARS-CoV-2 reagents for the BD MAX System (Becton Dickinson, Sparks, MD, USA), and the GenMark ePlex SARS-CoV-2 test (GenMark, Carlsbad, CA, USA). All diagnostic orders were placed by emergency clinicians who had EHR-embedded access to a previously validated clinical decision guideline for influenza testing^31^ and to SARS-CoV2 testing criteria that were regularly updated by our Department of Hospital Epidemiology and Infection Control based on guidance from the Centers for Disease Control and Prevention^32^. Secondary outcomes were hospitalization, intensive care unit (ICU) admission, and in-hospital mortality. Critical care used in secondary analyses was defined as a composite of ICU admission or in-hospital mortality. Data for these outcomes were limited to the index hospital encounter and were collected from the EHR as described above.

### Analysis

Continuous variables were expressed as median with interquartile range (IQR) and compared using the Mann–Whitney U test. Categorical variables were expressed as number and percentages and were compared using the χ2 test. Test performance was evaluated using binary classification measures. The area under the Receiver Operating Characteristics curve (AUC) was calculated using logistic regression models. MDW and other leukocyte parameters were modeled in isolation as continuous variables. Sensitivity, specificity, positive and negative predictive values, and likelihood ratios were calculated using previously established laboratory cutoffs with definitions for dichotomization as normal or abnormal. All analyses were performed in Python Version 3.

## Results

**Figure 1:**
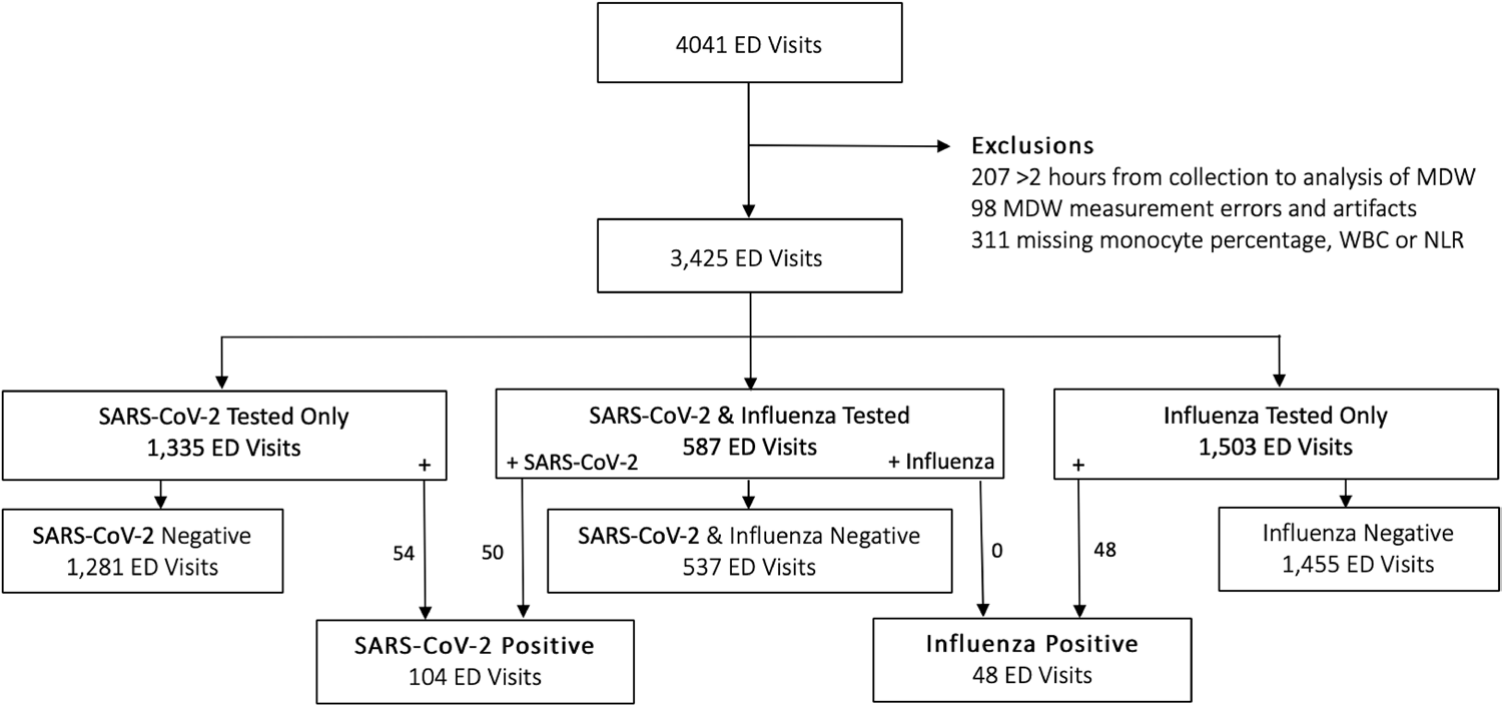
Study Flow Diagram. Abbreviations: ED, Emergency Department; MDW, monocyte distribution width; WBC, white blood cell count; NLR, neutrophil to lymphocyte ratio; SARS-CoV-2, severe acute respiratory syndrome coronavirus 2.

A total of 3,425 ED visits were included in the final cohort. Diagnostic testing for SARS-CoV-2 was performed on 1,922 visits with 104 (5.4%) testing positive (Fig. 1). In a total of 2090 ED visits testing for influenza was performed, with 48 (2.3%) testing positive. Of the total cohort, 587 visits received diagnostic testing for both SARS-CoV-2 and influenza; no patients tested positive for both viruses.

### Study cohort

Characteristics of the study cohort grouped by test and positivity may be seen in Table 1. For the group tested for SARS-CoV-2, the median patient age was 54 years, 51.3% were female and 71.0% were African American. The group tested for influenza was similar, with a median age of 53 years, 54.0% were female and 60.8% were African American. Common presenting complaints for both SARS-CoV-2 and influenza positive patients were shortness of breath and fever. However, chest pain was more prevalent in SARS-CoV-2 positive patients. Hypertension, kidney disease, and diabetes were the most common co-morbidities in SARS-CoV-2 positive patients. Hypertension, liver disease, and immunosuppression were most common in the influenza positive group (Table 1). Patients positive for SARS-CoV-2 demonstrated higher severity of illness compared to influenza. In the SARS-CoV-2 positive patient group, 42.3% required hospital admission, 2.9% required ICU admission and 1.0% died in-hospital. In the influenza positive patient group, 22.9% required hospital admission, 6.3% required ICU admission, and none died in-hospital.

**Table 1:** Characteristics of study participants stratified by SARS-CoV-2 and influenza infection status.

	SARS-CoV-2			Influenza		
	Tested	Positive	Negative	Tested	Positive	Negative
**ED Visits, N**	1922	104	1818	2090	48	2042
**Demographics**						
Age, median (IQR)	54.0 (39.0–64.0)	55.0 (39.0–63.0)	54.0 (39.0–65.0)	53.0 (36.0–64.0)	49.5 (28.8–58.5)	53.0 (36.0–65.0)
Gender Female, N (%)	986 (51.3%)	57 (54.8%)	929 (51.1%)	1129 (54.0%)	25 (52.1%)	1104 (54.1%)
Black, N (%)	1365 (71.0%)	77 (74.0%)	1288 (70.8%)	1271 (60.8%)	38 (79.2%)	1233 (60.4%)
White, N (%)	439 (22.8%)	13 (12.5%)	426 (23.4%)	611 (29.2%)	4 (8.3%)	607 (29.7%)
Other, N (%)	119 (6.2%)	14 (13.5%)	105 (5.8%)	208 (10.0%)	6 (12.5%)	202 (9.9%)
Ethnicity Hispanic/Latino, N (%)	91 (4.7%)	11 (10.6%)	80 (4.4%)	139 (6.7%)	2 (4.2%)	137 (6.7%)
**Complaints**, N (%)						
Shortness of Breath	227 (11.8%)	26 (25.0%)	201 (11.1%)	298 (14.3%)	7 (14.6%)	291 (14.3%)
Chest Pain	229 (11.9%)	14 (13.5%)	215 (11.8%)	216 (10.3%)	3 (6.2%)	213 (10.4%)
Abdominal Pain	152 (7.9%)	4 (3.8%)	148 (8.1%)	158 (7.6%)	4 (8.3%)	154 (7.5%)
Headache	52 (2.7%)	4 (3.8%)	48 (2.6%)	87 (4.2%)	0 (0.0%)	87 (4.3%)
Emesis	49 (2.5%)	1 (1.0%)	48 (2.6%)	84 (4.0%)	0 (0.0%)	84 (4.1%)
Fever	41 (2.1%)	7 (6.7%)	34 (1.9%)	69 (3.3%)	5 (10.4%)	64 (3.1%)
**Co-Morbidities**, N (%)						
Coronary Artery Disease	293.0 (15.2%)	14.0 (13.5%)	279.0 (15.3%)	193.0 (9.2%)	3.0 (6.2%)	190.0 (9.3%)
Cancer	351.0 (18.3%)	22.0 (21.2%)	329.0 (18.1%)	350.0 (16.7%)	7.0 (14.6%)	343.0 (16.8%)
Cerebrovascular Disease	184.0 (9.6%)	8.0 (7.7%)	176.0 (9.7%)	127.0 (6.1%)	1.0 (2.1%)	126.0 (6.2%)
Diabetes	432.0 (22.5%)	28.0 (26.9%)	404.0 (22.2%)	311.0 (14.9%)	5.0 (10.4%)	306.0 (15.0%)
Heart Failure	317.0 (16.5%)	12.0 (11.5%)	305.0 (16.8%)	184.0 (8.8%)	3.0 (6.2%)	181.0 (8.9%)
Hypertension	815.0 (42.4%)	48.0 (46.2%)	767.0 (42.2%)	608.0 (29.1%)	12.0 (25.0%)	596.0 (29.2%)
Immunosuppression	408.0 (21.2%)	18.0 (17.3%)	390.0 (21.4%)	319.0 (15.3%)	10.0 (20.8%)	309.0 (15.1%)
Kidney Disease	495.0 (25.7%)	33.0 (31.7%)	462.0 (25.4%)	328.0 (15.7%)	5.0 (10.4%)	323.0 (15.8%)
Liver Disease	409.0 (21.3%)	14.0 (13.5%)	395.0 (21.7%)	323.0 (15.5%)	12.0 (25.0%)	311.0 (15.2%)
Prior Respiratory Failure	22.0 (1.1%)	2.0 (1.9%)	20.0 (1.1%)	20.0 (1.0%)	0.0 (0.0%)	20.0 (1.0%)
**Severity of Illness**						
In-Hospital Mortality, N (%)	18.0 (0.9%)	1.0 (1.0%)	17.0 (0.9%)	26.0 (1.2%)	0.0 (0.0%)	26.0 (1.3%)
ICU Admission, N (%)	48 (2.5%)	3 (2.9%)	45 (2.5%)	100 (4.8%)	3 (6.3%)	97 (4.8%)
Inpatient Admission, N (%)	842 (43.8%)	44 (42.3%)	798 (43.9%)	776 (37.1%)	11 (22.9%)	765 (37.5%)
Hospital LOS hours, median (IQR)	41.9 (11.8–127.0)	33.4 (11.4–133.9)	45.4 (11.8–127.0)	27.8 (10.4–105.7)	14.8 (6.8–54.5)	28.1 (10.5–108.2)
**CBC Parameters**						
MDW (units), median (IQR)	19.1 (17.5–21.0)	23.0 (20.5–25.1)	18.9 (17.4–20.7)	19.2 (17.4–21.8)	24.1 (22.0–26.9)	19.1 (17.4–21.6)
Monocyte % (IQR)	7.6 (5.9–9.6)	9.4 (6.8–13.2)	7.5 (5.9–9.5)	7.4 (5.8–9.5)	9.2 (6.6–11.6)	7.4 (5.8–9.4)
WBC (× 10^9^/L), median (IQR)	7.2 (5.4–9.7)	5.5 (4.1–7.3)	7.3 (5.6–9.8)	7.4 (5.6–9.9)	5.8 (4.4–7.5)	7.4 (5.6–10.0)
NLR, median (IQR)	3.0 (1.9–5.6)	2.9 (1.9–5.8)	3.0 (1.9–5.5)	3.4 (2.1–6.6)	4.1 (1.9–10.7)	3.4 (2.1–6.6)

Unless otherwise noted, values are N (%).

*SARS-CoV-2* severe acute respiratory syndrome coronavirus 2, *ED* emergency department, *IQR* interquartile range, *ICU* intensive care unit, *LOS* length of stay, *CBC* complete blood count, *MDW* monocyte distribution width, *WBC* white blood cell count, *NLR* neutrophil to lymphocyte ratio.

### Accuracy of MDW, monocyte percentage, WBC and NLR for SARS-CoV-2 and influenza infection

**Figure 2:**
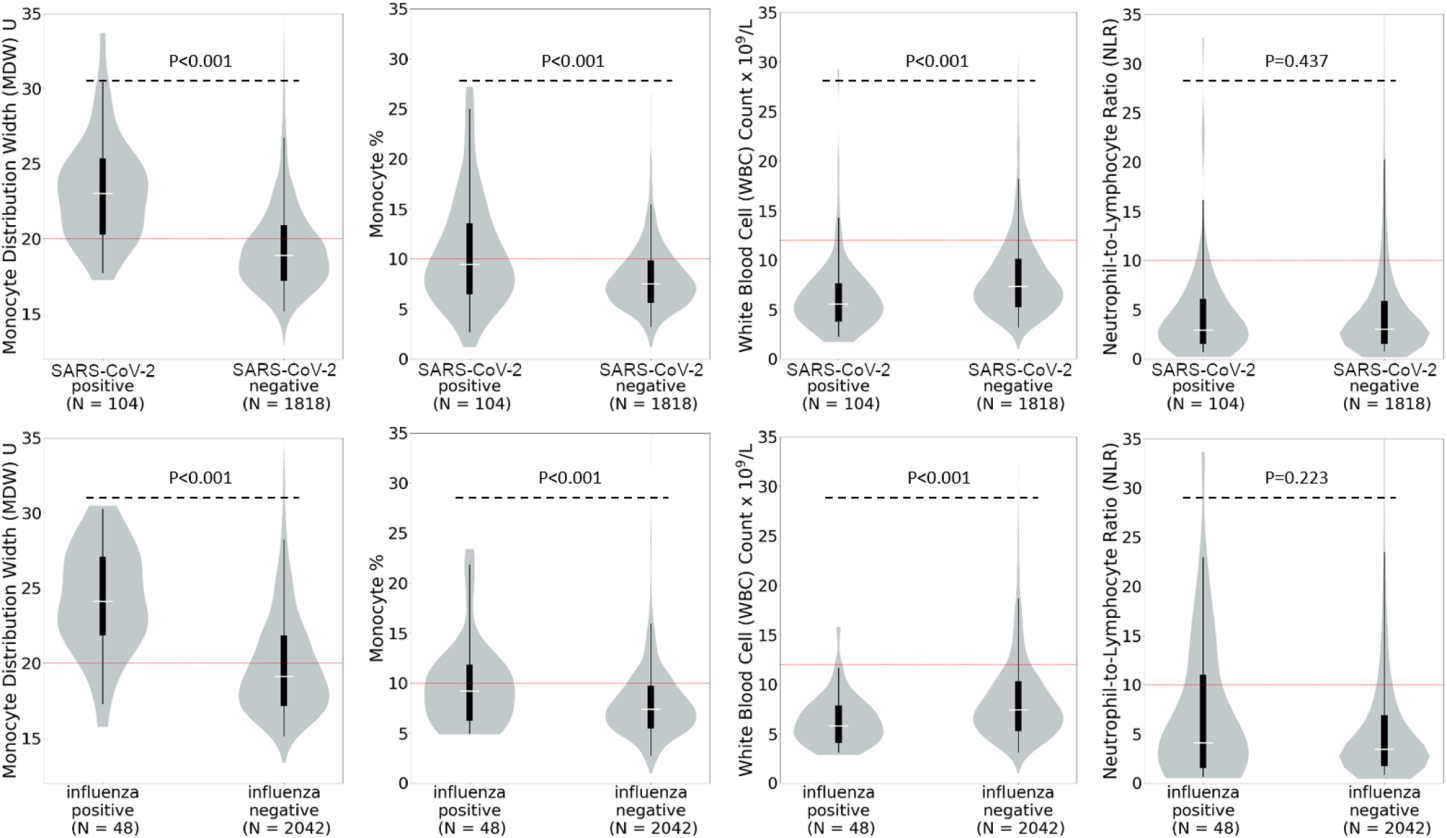
Monocyte Distribution Width (MDW), Monocyte Percentage, White Blood Cell count (WBC), and Neutrophil-to-Lymphocyte ratio in SARS-CoV-2 and Influenza Positive and Negative Patients. The red dotted line represents the normal upper limit for each parameter. Median MDW is above the normal cutoff in patients who tested positive for severe acute respiratory syndrome coronavirus 2 (SARS-CoV-2) and influenza but below this cutoff in patients who tested negative. Median monocyte percentage, WBC and NLR values were all within the normal range regardless of infection with SARS-CoV-2 or influenza.

MDW levels were significantly higher in patients who tested positive for SARS-CoV-2 (median 23.0U, IQR 20.5–25.1U) than in those who tested negative (18.9U, IQR 17.4–20.7U, *P* < 0.001) as seen in Fig. 2. Findings were similar for influenza, with a median MDW of 24.1U (IQR 22.0–26.9U) observed for influenza positive patients, compared with 19.1U (IQR 17.4–21.6U, *P*< 0.001) for influenza negative patients. The group of influenza negative patients in this cohort included 50 patients who tested positive for SARS-CoV-2. As patients with SARS-CoV-2 infection had elevated MDW values, this may have caused a slight bias towards a higher median MDW value in influenza negative patients. Despite this, the median MDW value in the influenza negative group was still below the cutoff of 20U and the difference between patients who tested positive and those who tested negative for influenza was still significant (*P* < 0.001). (This did not occur in SARS-CoV-2 negative patients as none of these patients tested positive for influenza.) Median monocyte percentage and WBC demonstrated smaller differences (*P*< 0.001) in positive versus negative patients for both viruses, however, values for positive and negative patients were within the accepted normal ranges for these parameters. These trends were not seen for NLR, which was similar for groups with and without viral infection (Table 1 and Fig. 2).

**Figure 3:**
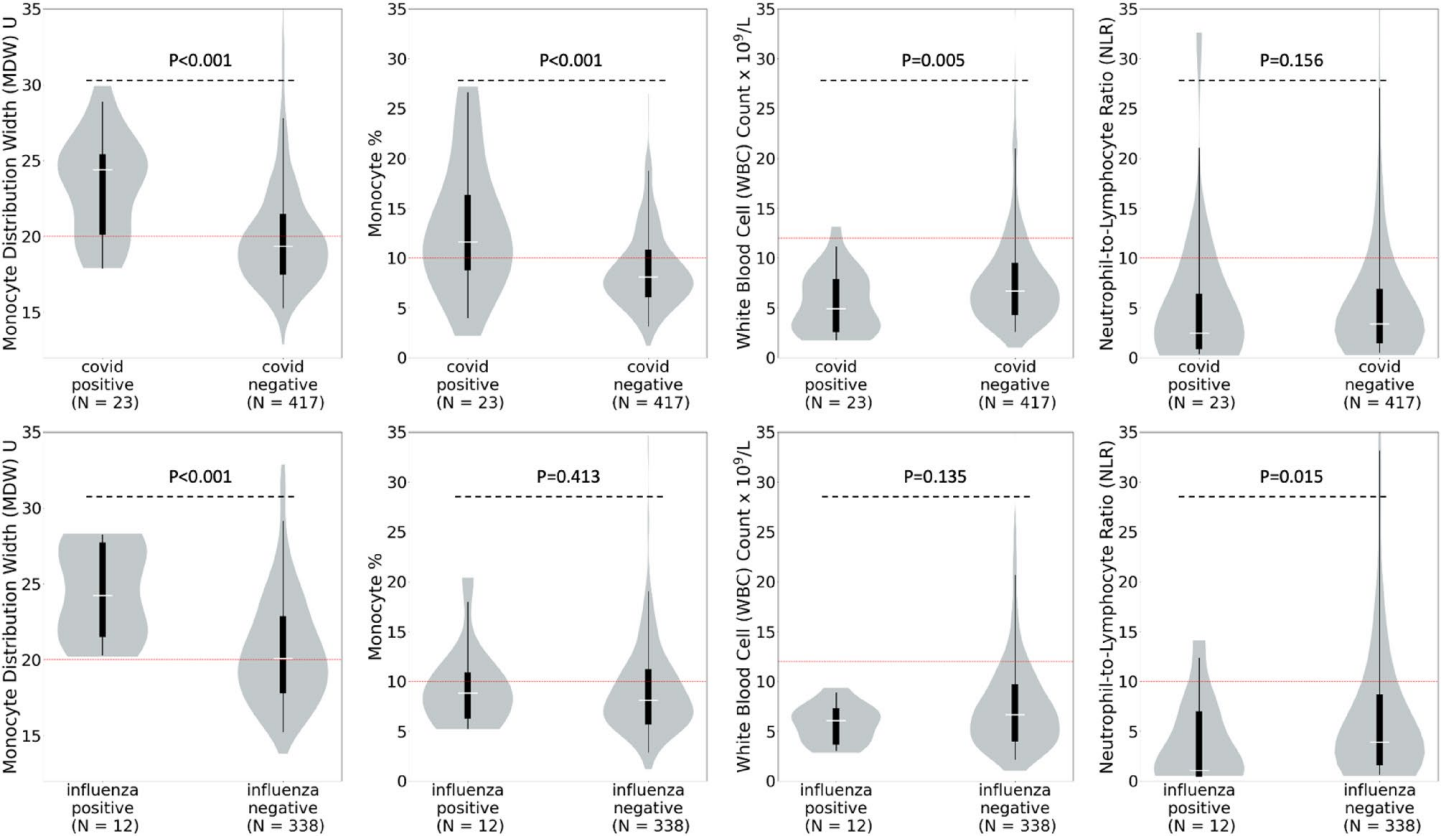
Monocyte Distribution Width (MDW), Monocyte Percentage, White Blood Cell count (WBC), and Neutrophil-to-Lymphocyte ratio in SARS-CoV-2 and Influenza Positive and Negative Immunocompromised Patients. A subgroup analysis was performed to evaluate the performance of each study parameter in immunocompromised patients. The red dotted line represents the normal upper limit for each parameter. Median MDW is above the normal cutoff in patients who tested positive for severe acute respiratory syndrome coronavirus 2 (SARS-CoV-2) and influenza but at or below this cutoff in patients who tested negative. Median monocyte percentage was above the normal range in immunocompromised patients who tested positive for SARS-CoV-2 but not for patients who tested positive for influenza. Median WBC and NLR values were within the normal range regardless of infection with SARS-CoV-2 or influenza.

In immunocompromised patients, MDW values were significantly elevated in patients who tested positive for SARS-CoV-2 (median 24.4U, IQR 20.3–25.2U) compared to those who tested negative (median 19.3U, IQR 17.7–21.3U, *P*< 0.001), and similarly elevated in those who tested positive for influenza (24.2U, IQR 21.7–27.5U) compared to those who tested negative (median 20.1U, IQR 18.0–22.7U). MDW levels were above the normal cutoff of 20U for patients with SARS-CoV-2 or influenza infections and at or below that number for those who tested negative. Of the other parameters evaluated (monocyte percentage, WBC and NLR), all except monocyte percentage had median values below accepted cutoffs regardless of infection with SARS-CoV-2 or influenza. These results indicate that MDW is also differentially elevated in immunocompromised patients with SARS-CoV-2 or influenza infections compared to those without these infections (Supplemental Table 1 and Fig. 3).

Overall accuracy (area under the Receiver Operating Characteristics curve [AUC]) of MDW was 0.83 (95% CI 0.79–0.86) for SARS-CoV-2 infection and 0.83 (95% CI 0.77–0.88) for influenza infection (Table 2). In contrast, the AUC for monocyte percentage, WBC count and NLR were below 0.70 for both viruses. At the established cutoff of 20, MDW sensitivity was 83.7% (95% CI 76.5–90.8) for SARS-CoV-2 and 89.6% (95% CI 80.9–98.2) for influenza, compared with sensitivities below 50% for percent monocyte, WBC, and NLR. MDW negative predictive values were 98.6 (95% CI 98.0–99.3) for SARS-CoV-2 infection and 99.6 (95% CI 99.3–100.0) for influenza. Negative likelihood ratios of 0.24 and 0.17 were achieved for SARS-CoV-2 and influenza, respectively, translating to an approximate four-to-six-fold decrease in odds of having a viral infection with an MDW value below 20U (Table 2).

**Table 2:** Test characteristics of the complete blood count (CBC) parameters evaluated.

	Abnormal Definition	AUC (95% CI)	Sensitivity % (95% CI)	Specificity % (95% CI)	PPV (95% CI)	NPV (95% CI)	+ LR	− LR
**SARS-CoV-2 Positive**								
MDW	> 20	0.83 (0.79–0.86)	83.7 (76.5–90.8)	67.8 (65.6–69.9)	12.9 (10.4–15.5)	98.6 (98.0–99.3)	2.60	0.24
Mono %	> 10	0.64 (0.58–0.70)	27.9 (19.3–36.5)	79.8 (77.9–81.6)	7.3 (4.8–9.9)	95.1 (94.0–96.2)	1.38	0.90
WBC	> 12	0.68 (0.63–0.73)	30.8 (21.9–39.6)	71.1 (69.0–73.2)	5.8 (3.8–7.7)	94.7 (93.5–95.9)	1.07	0.97
NLR	> 10	0.50 (0.45–0.56)	42.3 (32.8–51.8)	79.3 (77.4–81.1)	10.5 (7.5–13.4)	96.0 (95.0–97.0)	2.04	0.73
**Influenza Positive**								
MDW	> 20	0.83 (0.77–0.88)	89.6 (80.9–98.2)	61.1 (59.0–63.2)	5.1 (3.6–6.6)	99.6 (99.3–100.0)	2.30	0.17
Mono %	> 10	0.63 (0.55–0.72)	20.8 (9.3–32.3)	79.6 (77.8–81.3)	2.3 (0.9–3.8)	97.7 (97.0–98.4)	1.02	0.99
WBC	> 12	0.67 (0.60–0.74)	43.8 (29.7–57.8)	66.3 (64.2–68.3)	3.0 (1.7–4.2)	98.0 (97.3–98.8)	1.30	0.85
NLR	> 10	0.53 (0.43–0.63)	43.8 (29.7–57.8)	79.3 (77.5–81.0)	4.7 (2.8–6.7)	98.4 (97.7–99.0)	2.11	0.71

*SARS-CoV-2* severe acute respiratory syndrome coronavirus 2, *CBC* complete blood count, *MDW* monocyte distribution width, *WBC* white blood cell count, *NLR* neutrophil to lymphocyte ratio, *AUC* area under the Receiver Operating Characteristic curve, *CI* confidence interval, *PPV* positive predictive value, *NPV* negative predictive value, + *LR* positive likelihood ratio, − *LR* negative likelihood ratio.

**Figure 4:**
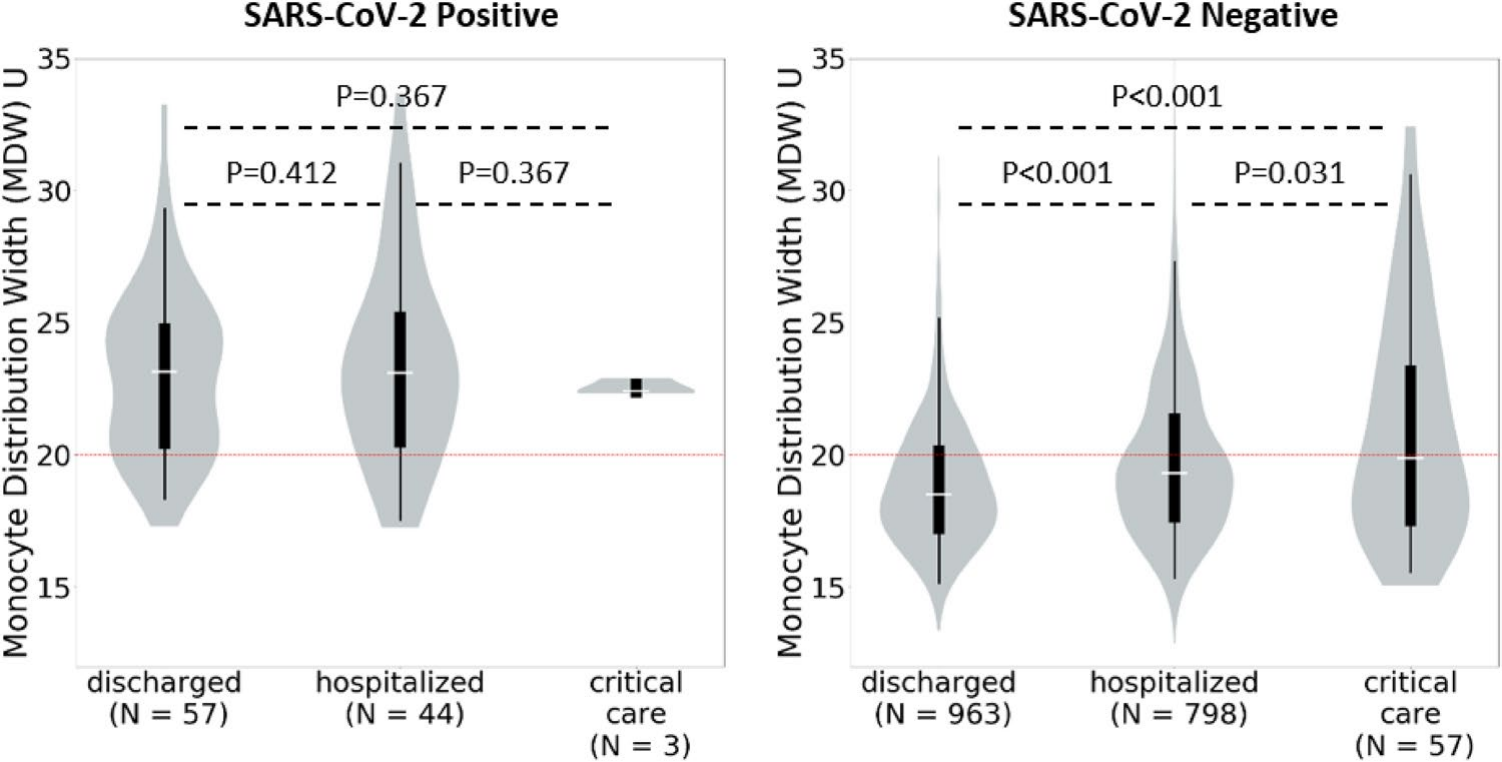
Monocyte Distribution Width (MDW) in SARS-CoV-2 Positive and Negative Patients Stratified by Illness Severity. The red dotted line represents the normal upper limit for each parameter. Median monocyte distribution width (MDW) is above the normal cutoff in patients who tested positive for severe acute respiratory syndrome coronavirus 2 (SARS-CoV-2) regardless of illness severity but below this cutoff in patients who tested negative with the exception of those who required intensive care unit (ICU) admission. MDW increased with increasing illness severity in SARS-CoV-2 negative patients.

**Figure 5:**
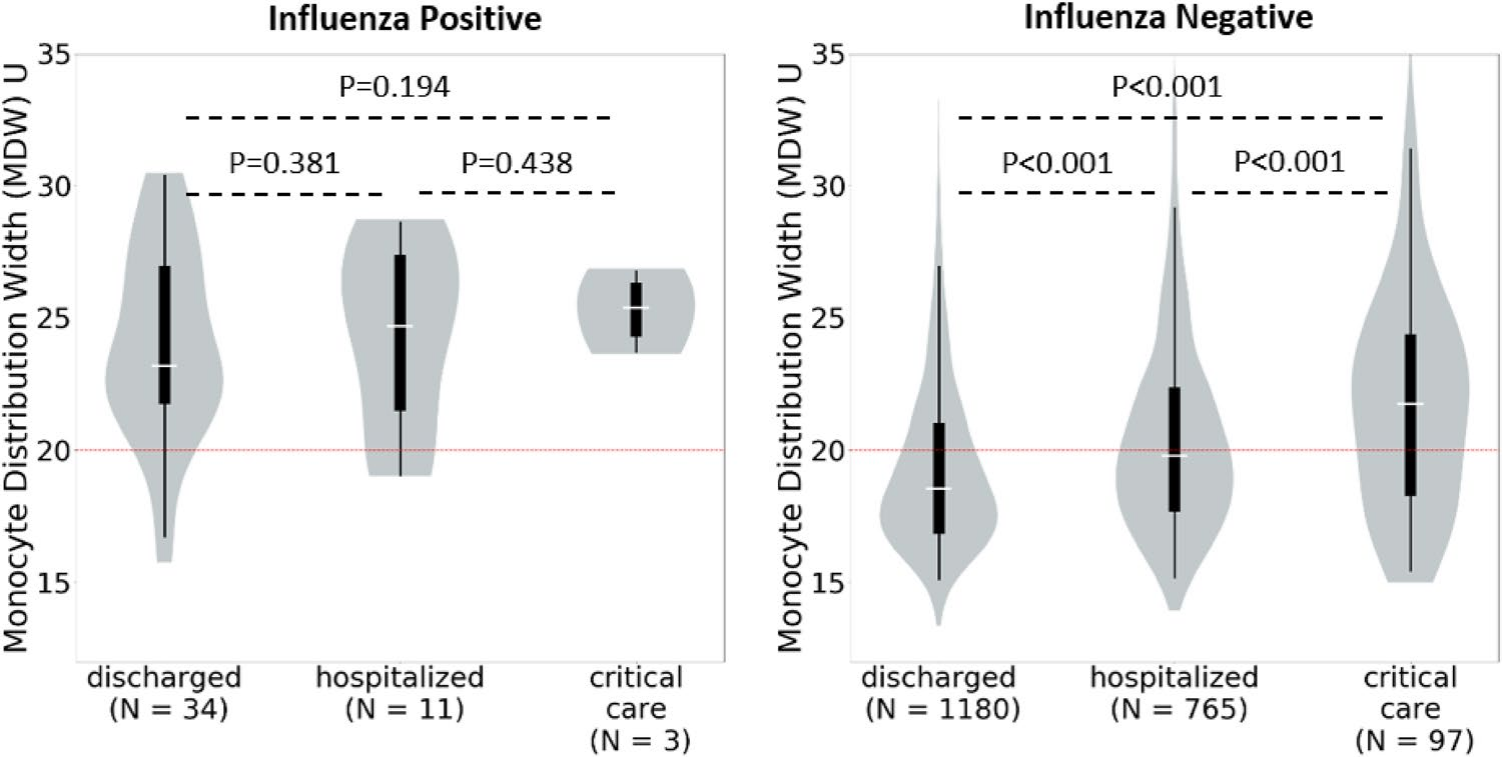
Monocyte Distribution Width (MDW) in Influenza Positive and Negative Patients Stratified by Illness Severity. The red dotted line represents the normal upper limit for each parameter. Median monocyte distribution width (MDW) is above the normal cutoff in patients who tested positive for influenza regardless of illness severity but below this cutoff in patients who tested negative with the exception of those who were hospitalized or required intensive care unit (ICU) admission. MDW increased with increasing illness severity in influenza negative patients.

As shown in Figs. 4 and 5, MDW was elevated for all patients with a viral infection, regardless of illness severity. No significant differences in MDW levels were seen between virally infected patients who were well enough to be discharged home after ED evaluation and those who required hospital ward or critical care admission (SARS-CoV-2, *P* > 0.05 and influenza, *P*  > 0.05, Figs. 4 and 5). These findings contrast with those observed for uninfected patients, in whom MDW values were correlated with severity of illness. As shown in Figs. 4 and 5, median MDW was below the established cutoff of 20U in uninfected patients who were discharged home after ED visit but was elevated in those with critical illness requiring ICU admission (*P* < 0.001).

## Discussion

In this study we found that MDW, a measure of monocyte activation that can be routinely reported on the CBC differential, has potential to be used as an ED-based screening test for respiratory viral infection. Overall accuracy (AUC) of MDW for both SARS-CoV-2 and influenza infection was very good; high sensitivities and negative predictive values were also achieved for both viruses using the previously established and currently reported cut-off for MDW. The identification of such a measure embedded within a laboratory panel that is routinely ordered in acute care settings is a major advancement^33,34^. It affords the opportunity for the development and implementation of broad-based screening programs to enable rapid identification and isolation of patients with contagious viral infections, even for cases that are unsuspected. Negative likelihood ratios achieved by MDW also suggest it could be used to preserve more expensive molecular diagnostics under conditions where testing resources are limited, as has been the case for SARS-CoV-2^18,19^. Importantly, the performance of MDW was unique among all leukocyte parameters examined, further substantiating its independent value for decision-making in acute care environments.

Multiple recent studies have illustrated that MDW is a useful biomarker for early detection of sepsis, and several have found that MDW levels increase in parallel with sepsis severity^1–3^. This includes a published analysis of the performance of MDW and other WBC parameters for identification of sepsis in our same patient cohort^3^. In the current study, we found that MDW is also elevated in the setting of SARS-CoV-2 and influenza infection, and that MDW elevation in this context is independent of illness severity; virally infected patients well enough to be discharged home had MDW values similar to those who required hospitalization or ICU admission. In our cohort, MDW did track linearly with disease severity as reported by others^1–3^, but only in patients without SARS-CoV-2 or influenza infection. These findings suggest that infections with SARS-CoV-2 or influenza may be confounders when MDW is utilized as a screening tool for sepsis; an elevated MDW could be due to a viral infection with a less severe prognosis. In practice, elevated MDW should likely prompt consideration of, or investigation for, SARS-CoV-2 or influenza infection prior to, or in parallel with, activation of protocols for sepsis diagnosis and treatment.

While a small number of other studies have investigated the association between MDW and SARS-CoV-2 infection^35,36^, our study is the largest to compare MDW values in patients with and without SARS-CoV-2 infections. Our findings are supported by the previous reports which suggest that MDW is elevated in patients with SARS-CoV-2 infection compared to those not infected with this virus^35,36^ with mean differences between the two groups ranging from 1.8 to 7.2 U. In a pooled analysis of three available studies, Lippi et al. determined that the weighted mean difference in the MDW for patients with SARS-CoV-2 compared to patients without this virus was 3.95U (95% CI 1.41–6.49 U)^36^. In our study, this difference was 4.2U. Collectively, these data strongly suggest MDW is a reliable marker of SARS-CoV-2 infection.

Our study is also one of very few (and the largest to date) to have investigated MDW for identification of influenza infections. In the only other currently published report on MDW and influenza, Lin et al. evaluated the performance of MDW in 174 ED patients with suspected respiratory infection who were also subjected to molecular viral testing and hospitalized after their ED encounter. Nine patients in this population tested positive for SARS-CoV-2 and 24 for influenza; MDW was differentially elevated in patients with either viral infection. In this small study, discriminatory performance of MDW was moderate for both SARS-CoV-2 and influenza, with reported AUCs of 0.70 and 0.63, respectively^35^. Our study, performed in a much larger and less selected population, achieved much better performance. The AUC of 0.83 for both SARS-CoV-2 and influenza observed here, along with the binary classification measures shown in Table 2, suggest MDW may be an effective pragmatic, ED population-wide screening test for these viral infections.

The performance of MDW as a screen for SARS-CoV-2 and influenza infection reported here is comparable to that of many currently approved, more expensive, and less routinely available tests. While RT-PCR is the gold standard for diagnosis of these viral infections, several other platforms play important roles in their management^19,37^. SARS-CoV-2 antigen-based tests are routinely used in outpatient and urgent care settings and have been proposed as a useful adjunct to RT-PCR in the ED. The sensitivity of MDW for SARS-CoV-2 infection is comparable to that of many of these antigen-based SARS-CoV-2 testing platforms^38–40^. In our study, MDW was also as sensitive for influenza infection as several CLIA-waivered rapid influenza detection tests currently used in outpatient and ED settings^41,42^. It is important to note, however, that the aforementioned rapid antigen tests are particularly sensitive for contagious cases of SARS-CoV-2 infection. While our study was not designed to evaluate contagion, our finding that such a level of performance can be achieved using a component of the CBC differential, (a panel that is routinely ordered regardless of clinical suspicion for viral infection) is unique and potentially practice changing.

## Limitations

Our study does have limitations that should be considered. First, while overall performance (AUC) is independent of threshold, other performance parameters (sensitivity, specificity, predictive values, and likelihood ratios) were affected by our decision to employ the previously established cut-off of 20U for MDW. This cutoff was established for early detection of sepsis and was approved by the Food and Drug Administration (FDA). However, this may not be the optimal cut-off for viral detection under all scenarios. Clinician leaders may select different cut-offs to achieve optimal sensitivity and specificity for specific contexts, such as periods when isolation of potential viral infections is deemed critically important or when preservation of molecular tests is paramount (see Supplemental Fig. 1 for performance measures over a range of cut-offs). Second, the observational design of this study precluded strict control of diagnostic testing criteria. Many patients were tested for either influenza or SARS-CoV-2 and not both, and it is possible that some patients tested for one viral infection could have had an undetected infection by the other. We expect such cases were rare however, as all isolated influenza testing was performed prior to the first case of SARS-CoV-2 detected in our health system and isolated SARS-CoV-2 testing was guided by surveillance indicating very low burden of circulating influenza. In the subset tested for both viruses, no cases of co-infection were identified. Third, our analysis was limited to SARS-CoV-2 and influenza. While these are two of the most important respiratory infections in modern medicine, we are unable to determine whether MDW is also elevated in the setting of other less common respiratory viral infections using the data analyzed here. Finally, this was a single site study, which may limit its generalizability. However, our sample size was very large with more than 3500 ED visits and 160 viral infections detected. While external validation will be necessary, our findings are in agreement with those of previous, smaller studies.

## Conclusions

In conclusion, MDW is differentially elevated in SARS-CoV-2 and influenza infections and may be an effective tool for broad-based screening for these infections in healthcare settings, allowing for the identification of patients who may benefit from confirmatory viral testing. Further evaluation of the clinical utility of MDW-based viral infection screening programs is an important future aim. Current programs that employ MDW for early detection of sepsis should consider viral infection in patients with elevated MDW who do not have clear alternative sources of infection or signs of severe disease.

## Supplementary Information

Supplementary Information.

## Data Availability

The datasets generated during and analyzed during the current study are available from the corresponding author on reasonable request.
