# Supplementary material for: Monocyte distribution width as a pragmatic screen for SARS-CoV-2 or influenza infection
Source: Sci Rep. 2022 Dec 13;12:21528. doi: 10.1038/s41598-022-24978-w (PMC9745720; doi:10.1038/s41598-022-24978-w)
Supplement: Supplementary file 1 — Supplementary Information. [file 41598_2022_24978_MOESM1_ESM.docx]

**Supplemental Table 1. Characteristics of Immunocompromised Study Participants Stratified by SARS-CoV-2 and Influenza Infection Status**

|  | **SARS-CoV-2** | | | **Influenza** | | |
| --- | --- | --- | --- | --- | --- | --- |
|  | **Tested** | **Positive** | **Negative** | **Tested** | **Positive** | **Negative** |
| **ED Visits, N** | 440 | 23 | 417 | 350 | 12 | 338 |
| **Demographics** |  |  |  |  |  |  |
| Age, median (IQR) | 57.0 (45.0-66.0) | 55.0 (40.5-61.0) | 57.0 (47.0-66.0) | 57.0 (47.0-66.0) | 50.5 (33.5-56.5) | 57.5 (47.0-66.0) |
| Gender Female, N (%) | 201 (45.7%) | 11 (47.8%) | 190 (45.6%) | 170 (48.6%) | 7 (58.3%) | 163 (48.2%) |
| Black or African American, N (%) | 303 (68.9%) | 19 (82.6%) | 284 (68.1%) | 218 (62.3%) | 9 (75.0%) | 209 (61.8%) |
| White or Caucasian, N (%) | 114 (25.9%) | 2 (8.7%) | 112 (26.9%) | 108 (30.9%) | 1 (8.3%) | 107 (31.7%) |
| Other, N (%) | 23 (5.2%) | 2 (8.7%) | 21 (5.0%) | 24 (6.9%) | 2 (16.7%) | 22 (6.5%) |
| Ethnicity Hispanic/Latino, N (%) | 21 (4.8%) | 2 (8.7%) | 19 (4.6%) | 14 (4.0%) | 1 (8.3%) | 13 (3.8%) |
| **Complaints, N (%)** |  |  |  |  |  |  |
| Shortness of Breath | 52 (11.8%) | 8 (34.8%) | 44 (10.6%) | 68 (19.4%) | 1 (8.3%) | 67 (19.8%) |
| Chest Pain | 59 (13.4%) | 6 (26.1%) | 53 (12.7%) | 36 (10.3%) | 1 (8.3%) | 35 (10.4%) |
| Abdominal Pain | 36 (8.2%) | 0 (0.0%) | 36 (8.6%) | 17 (4.9%) | 2 (16.7%) | 15 (4.4%) |
| Headache | 9 (2.0%) | 1 (4.3%) | 8 (1.9%) | 12 (3.4%) | 0 (0.0%) | 12 (3.6%) |
| Emesis | 11 (2.5%) | 0 (0.0%) | 11 (2.6%) | 15 (4.3%) | 0 (0.0%) | 15 (4.4%) |
| Fever | 18 (4.1%) | 3 (13.0%) | 15 (3.6%) | 20 (5.7%) | 0 (0.0%) | 20 (5.9%) |
| **Co-Morbidities, N (%)** |  |  |  |  |  |  |
| Coronary Artery Disease | 82.0 (18.6%) | 5.0 (21.7%) | 77.0 (18.5%) | 56.0 (16.0%) | 1.0 (8.3%) | 55.0 (16.3%) |
| Cancer | 107.0 (24.3%) | 7.0 (30.4%) | 100.0 (24.0%) | 102.0 (29.1%) | 3.0 (25.0%) | 99.0 (29.3%) |
| Cerebrovascular Disease | 53.0 (12.0%) | 2.0 (8.7%) | 51.0 (12.2%) | 34.0 (9.7%) | 1.0 (8.3%) | 33.0 (9.8%) |
| Diabetes | 134.0 (30.5%) | 9.0 (39.1%) | 125.0 (30.0%) | 105.0 (30.0%) | 3.0 (25.0%) | 102.0 (30.2%) |
| Heart Failure | 93.0 (21.1%) | 4.0 (17.4%) | 89.0 (21.3%) | 49.0 (14.0%) | 2.0 (16.7%) | 47.0 (13.9%) |
| Hypertension | 257.0 (58.4%) | 15.0 (65.2%) | 242.0 (58.0%) | 187.0 (53.4%) | 6.0 (50.0%) | 181.0 (53.6%) |
| Immunosuppression | 407.0 (92.5%) | 17.0 (73.9%) | 390.0 (93.5%) | 319.0 (91.1%) | 10.0 (83.3%) | 309.0 (91.4%) |
| Kidney Disease | 219.0 (49.8%) | 13.0 (56.5%) | 206.0 (49.4%) | 156.0 (44.6%) | 3.0 (25.0%) | 153.0 (45.3%) |
| Liver Disease | 123.0 (28.0%) | 2.0 (8.7%) | 121.0 (29.0%) | 92.0 (26.3%) | 6.0 (50.0%) | 86.0 (25.4%) |
| Prior Respiratory Failure | 6.0 (1.4%) | 0.0 (0.0%) | 6.0 (1.4%) | 5.0 (1.4%) | 0.0 (0.0%) | 5.0 (1.5%) |
| **Severity of Illness** |  |  |  |  |  |  |
| In-Hospital Mortality, N (%) | 5.0 (1.1%) | 0.0 (0.0%) | 5.0 (1.2%) | 7.0 (2.0%) | 0.0 (0.0%) | 7.0 (2.1%) |
| Critical Care, N (%) | 13 (3.0%) | 0 (0.0%) | 13 (3.1%) | 23 (6.6%) | 0 (0.0%) | 23 (6.8%) |
| Hospital Admit, N (%) | 242 (55.0%) | 15 (65.2%) | 227 (54.4%) | 174 (49.7%) | 3 (25.0%) | 171 (50.6%) |
| Hospital Duration, Median, (IQR) | 76.3 (20.3-149.2) | 124.4 (33.0-202.1) | 76.2 (20.4-148.4) | 73.0 (16.5-148.5) | 17.7 (7.4-34.3) | 74.4 (17.8-149.2) |
| **CBC Parameters** |  |  |  |  |  |  |
| MDW, median (IQR) | 19.4 (17.7-21.6) | 24.4 (20.3-25.2) | 19.3 (17.7-21.3) | 20.2 (18.0-22.9) | 24.2 (21.7-27.5) | 20.1 (18.0-22.7) |
| Monocyte %, median (IQR) | 8.2 (6.4-10.7) | 11.6 (9.1-16.0) | 8.1 (6.4-10.5) | 8.1 (6.0-10.9) | 8.9 (6.6-10.6) | 8.1 (6.0-10.9) |
| WBC (x10^9^/L), median (IQR) | 6.6 (4.5-9.2) | 4.9 (2.9-7.6) | 6.7 (4.6-9.2) | 6.6 (4.3-9.3) | 6.1 (4.0-7.0) | 6.7 (4.3-9.4) |
| NLR, median (IQR) | 3.3 (1.7-6.6) | 2.5 (1.2-6.1) | 3.4 (1.8-6.6) | 3.8 (1.8-8.3) | 1.1 (0.7-6.7) | 3.9 (1.9-8.4) |

Unless otherwise noted, values are N (%). Abbreviations: SARS-CoV-2, severe acute respiratory syndrome coronavirus 2; ED, emergency department; IQR, interquartile range; ICU, intensive care unit; LOS, length of stay; CBC, complete blood count; MDW, monocyte distribution width; WBC, white blood cell count; NLR, neutrophil to lymphocyte ratio.


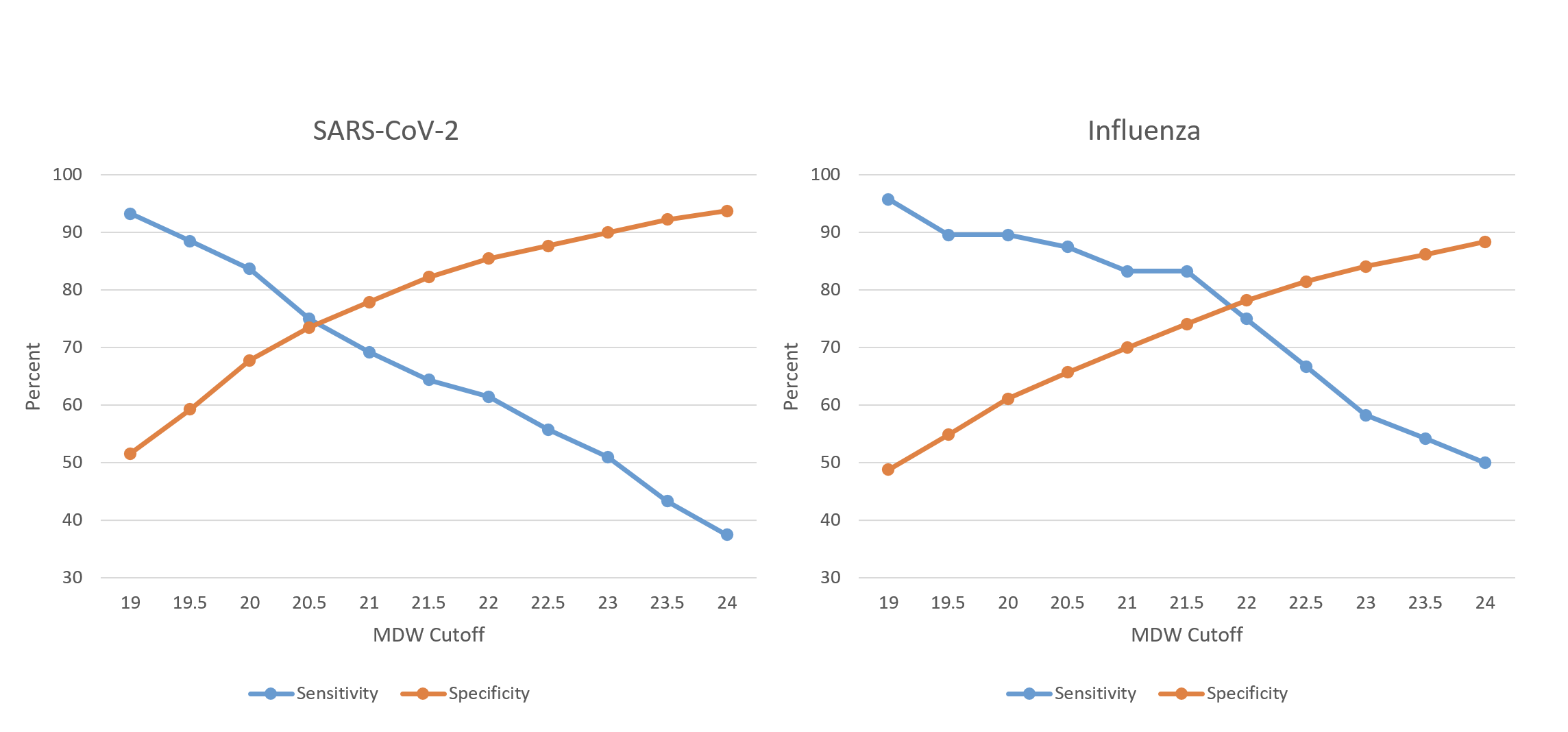
**Supplemental Figure 1. Sensitivities and Specificities for Monocyte Distribution Width (MDW) at Different Cutoffs**
